# The Mediating Roles of Medical Mistrust, Knowledge, Confidence and Complacency of Vaccines in the Pathways from Conspiracy Beliefs to Vaccine Hesitancy

**DOI:** 10.3390/vaccines9111342

**Published:** 2021-11-17

**Authors:** Xiaoning Zhang, Yuqing Guo, Qiong Zhou, Zaixiang Tan, Junli Cao

**Affiliations:** 1 School of Management, Xuzhou Medical University, Xuzhou 221004, China; 2 School of Nursing, Xuzhou Medical University, Xuzhou 221004, China; 300108110817@stu.xzhmu.edu.cn (Y.G.); 301910811587@stu.xzhmu.edu.cn (Q.Z.); 3 Jiangsu Province Key Laboratory of Anesthesiology, Xuzhou Medical University, Xuzhou 221004, China; caojl0310@aliyun.com; 4 Jiangsu Province Key Laboratory of Anesthesia and Analgesia Application Technology, Xuzhou Medical University, Xuzhou 221004, China

**Keywords:** vaccine hesitancy, conspiracy beliefs, pathways, confidence and complacency, medical mistrust

## Abstract

Background: Vaccine hesitancy, associated with medical mistrust, confidence, complacency and knowledge of vaccines, presents an obstacle to the campaign against the coronavirus disease 2019 (COVID-19). The relationship between vaccine hesitancy and conspiracy beliefs may be a key determinant of the success of vaccination campaigns. This study provides a conceptual framework to explain the impact of pathways from conspiracy beliefs to COVID-19 vaccine hesitancy with regard to medical mistrust, confidence, complacency and knowledge of vaccines. Methods: A non-probability study was conducted with 1015 respondents between 17 April and 28 May 2021. Conspiracy beliefs were measured using the coronavirus conspiracy scale of Coronavirus Explanations, Attitudes, and Narratives Survey (OCEANS), and vaccine conspiracy beliefs scale. Medical mistrust was measured using the Oxford trust in doctors and developers questionnaire, and attitudes to doctors and medicine scale. Vaccine confidence and complacency were measured using the Oxford COVID-19 vaccine confidence and complacency scale. Knowledge of vaccines was measured using the vaccination knowledge scale. Vaccine hesitancy was measured using the Oxford COVID-19 vaccine hesitancy scale. Confirmatory factor analysis (CFA) was used to evaluate the measurement models for conspiracy beliefs, medical mistrust, confidence, complacency, and knowledge of vaccines and vaccine hesitancy. The structural equation modeling (SEM) approach was used to analyze the direct and indirect pathways from conspiracy beliefs to vaccine hesitancy. Results: Of the 894 (88.1%) respondents who were willing to take the COVID-19 vaccine without any hesitancy, the model fit with the CFA models for conspiracy beliefs, medical mistrust, confidence, complacency and knowledge of vaccines, and vaccine hesitancy was deemed acceptable. Conspiracy beliefs had significant direct (*β* = 0.294), indirect (*β* = 0.423) and total (*β* = 0.717) effects on vaccine hesitancy; 41.0% of the total effect was direct, and 59.0% was indirect. Conspiracy beliefs significantly predicted vaccine hesitancy by medical mistrust (*β* = 0.210), confidence and complacency (*β* = 0.095), knowledge (*β* = 0.079) of vaccines, explaining 29.3, 11.0, and 13.2% of the total effects, respectively. Conspiracy beliefs significantly predicted vaccine hesitancy through the sequential mediation of knowledge of vaccines and medical mistrust (*β* = 0.016), explaining 2.2% of the total effects. Conspiracy beliefs significantly predicted vaccine hesitancy through the sequential mediation of confidence and complacency, and knowledge of vaccines (*β* = 0.023), explaining 3.2% of the total effects. The SEM approach indicated an acceptable model fit (χ^2^/df = 2.464, RMSEA = 0.038, SRMR = 0.050, CFI = 0.930, IFI = 0.930). Conclusions: The sample in this study showed lower vaccine hesitancy, and this study identified pathways from conspiracy beliefs to COVID-19 vaccine hesitancy in China. Conspiracy beliefs had direct and indirect effects on vaccine hesitancy, and the indirect association was determined through medical mistrust, confidence, complacency, and knowledge of vaccines. In addition, both direct and indirect pathways from conspiracy beliefs to vaccine hesitancy were identified as intervention targets to reduce COVID–19 vaccine hesitancy.

## 1. Introduction

The coronavirus disease 2019 (COVID-19) vaccine coverage potentially influenced the global control of the pandemic, mainly focusing on hospitalization and mortality reduction, and the societal and economic recovery, thus, governments must ensure the equitable distribution of a safe and effective COVID-19 vaccine [1]. Acceptance of the COVID-19 vaccine is crucial for achieving sufficient immunization coverage to end this global pandemic, but vaccine hesitancy, referring to the delay in acceptance or refusal of vaccines despite availability of vaccination services, is one of the top 10 global health threats [2], which currently presents a substantial obstacle to achieving vaccine coverage and herd immunity [1]. Individuals considered as vaccine hesitant include those who accept certain vaccines while refusing others, and those who delay or accept vaccines but have concerns [2]. COVID-19 vaccine hesitancy has effects on both the individual and the community by increasing the risk of infecting and transmitting COVID-19 [3].

Beliefs are plausible determinants of vaccine hesitancy [4]. General vaccine beliefs have been found to predict vaccine refusal in the United States [5]. Vaccine-related beliefs associated with vaccine hesitancy include the following [6]: (1) individuals believe they would still be infected even if the vaccine worked; (2) the speed at which the vaccines would be developed, affecting safety and efficacy; (3) the degree to which receiving the vaccine may be physically unpleasant to individuals; (4) and the extent to which individuals being vaccinated would feel being experimented on [7]. There is a causal relationship between conspiracy theories and reduced willingness to vaccinate [8]. Conspiracy theories have four characteristics [9]: (1) an event is not as it seems; (2) individuals believe that those in power are covering up; (3) acceptance only by a minority; and (4) lack of evidence [10]. There is a substantial relationship between conspiracy beliefs and vaccine hesitancy [11], and an earlier study also revealed the connection between COVID-19 vaccine hesitancy and conspiracy beliefs [12].

The coronavirus conspiracy beliefs are ascribed to individuals based on long-standing prejudices, which are associated with excessive medical mistrust [10], vaccine hesitancy and vaccine decision making [3]. Trust in COVID-19 vaccines as well as the institutions are the determinants of the success of vaccination efforts [13]; the medical mistrust can be conceptualized as a conspiracy mentality, including excessive skepticism, and concerns over the detailed information [10]. Heightened vaccine hesitancy is attributable to mistrust in science or medicine [14] and negative perceptions on healthcare experience in low- and middle-income countries (LMICs) [15]. Negative views of doctors, vaccine developers and healthcare experiences feed into the medical mistrust, which contributes to the need for chaos directed at societal structures in both high-and middle-income countries (HIMIC) as well as LMICs [12]. COVID-19 vaccine hesitancy among African Americans may be driven by mistrust in the medical establishment [16]; this vaccine hesitant group were reported to have higher mistrust in scientists and healthcare workers in the UK [17]. A lack of confidence in vaccines is exacerbated by the misunderstanding of how immunization works, mistrust of government, healthcare services, and speed of vaccine development [3].

The framework of vaccine hesitancy includes complacency, confidence and convenience [12]. Beliefs about whether vaccines provide freedom or restriction have been shown to be part of the collective importance, complacency and confidence in vaccine decision making [7]. When individuals have a lower perception of the need for a vaccination, it is called vaccination complacency, influenced by general health beliefs, such as efficacy of vaccination [18]. Altruistic intent and collective responsibility have been highlighted, and acceptance of vaccine is about the collective importance that vaccines can save lives, and if individuals do not accept vaccines, the society would be less safe [19]. Emphasizing collective importance rather than personal responsibility may link to greater change in individuals’ behaviours, thus prosocial behaviour may be self-rewarding and affect all behaviours [10]. The communication of public health knowledge emphasizes the different kinds of collective responsibility to be consistent with prosocial motives [20].

Individuals may disregard compliance with COVID-19 prevention guidelines from government agencies, which reveals the effects of misinformation [21]. The COVID-19 public information crisis needs to be highlighted as misinformation was highly prevalent [10]; individuals who believed that COVID-19 was less severe may have been less willing to accept vaccination in LMICs [15]. Vaccine hesitancy in HIMIC is currently driven by misinformation, which may explain the rise of delayed vaccination in the United States, and the ‘cultural epidemic’ in Europe [22]. Exposure to COVID-19 and vaccine-related misinformation may reduce the proportion of individuals who choose to ‘definitely’ be vaccinated [23]. Individual protection against COVID-19, while concern about side effects, has been the main reason for vaccine hesitancy in both HIMIC [24] and LMICs [15]. The prevalence of vaccine hesitancy among Arabs is 80%, due mainly to side effects in healthcare policies [25]. Hesitant individuals mainly concerned about COVID-19 vaccine safety were reported to believe that vaccine development was rushed and thus unsafe [26]. Hesitant individuals viewed themselves with a degree of perception of vulnerability [20], but sociodemographic did not explain vaccine hesitancy to any significant extent [3].

**Figure 1:**
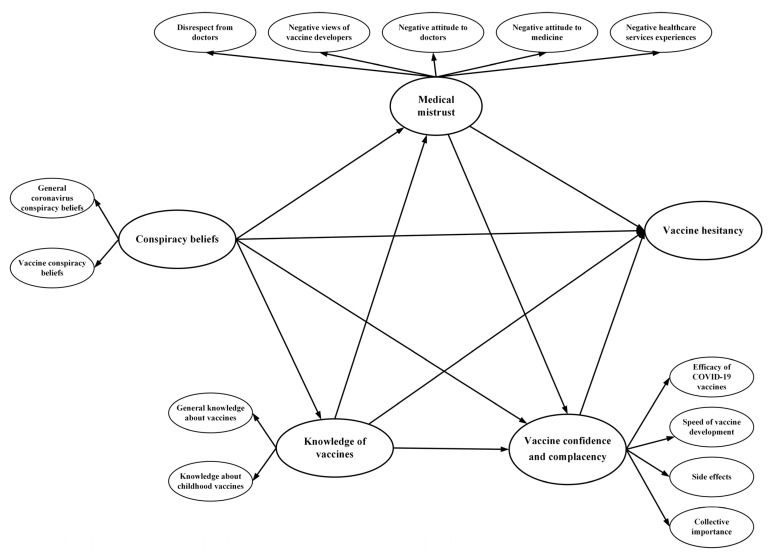
Hypothesized pathways from conspiracy beliefs to vaccine hesitancy of COVID-19 vaccines in the conceptual model.

Understanding the COVID-19 vaccine hesitancy is of global interest, a lag in vaccination may result in the emergence and spread of new virus variants that can overcome the immunity conferred by vaccinations [27]. Concerns that arise around vaccination campaigns are often case- and context-specific, making it difficult to predict how COVID-19 vaccines would be received in any given setting [15]. Many studies have been conducted on COVID-19 vaccine hesitancy in HIMIC, but few in LMICs; this study used metrics and indices to measure COVID-19 vaccine hesitancy [3]. Vaccine hesitancy has previously been found to be associated with medical mistrust, knowledge, confidence and complacency of vaccines. By using the structural equation modeling (SEM) approach, this study provides a conceptual framework to explain how the pathways from conspiracy beliefs to COVID-19 vaccine hesitancy (Figure 1), with regards to knowledge, confidence and complacency of vaccines, and medical mistrust: (1) a direct effect may exist between conspiracy beliefs and vaccine hesitancy; (2) conspiracy beliefs may indirectly influence vaccine hesitancy through medical mistrust, knowledge of vaccines, and vaccine confidence and complacency; (3) conspiracy beliefs may predict vaccine hesitancy through the sequential mediation of medical mistrust, and vaccine confidence and complacency; (4) conspiracy beliefs may predict vaccine hesitancy through knowledge of vaccines, and medical mistrust; and (5) conspiracy beliefs may predict vaccine hesitancy through knowledge of vaccines, medical mistrust, and vaccine confidence and complacency.

## 2. Methods

### 2.1. Study Design and Respondents

This non-probability study was conducted between 17 April and 28 May 2021 and respondents were quota sampled to represent the population for sex, age, income, and region in the community hospitals in Xuzhou, China. A total of 1015 respondents older than 18 years of age were recruited to complete this online investigation through a social media platform (WeChat), including: (1) sociodemographic characteristics; (2) conspiracy beliefs; (3) medical mistrust; (4) vaccine confidence and complacency; (5) knowledge of vaccines; and (6) vaccine hesitancy.

### 2.2. Sociodemographic Characteristics

Sociodemographic characteristics were collected including sex (male, female), age (18–29, 30–39, 40–49, ≥50 years), area of residence (rural, urban), marital status (single, married, others), educational level (middle school and below, senior high school, bachelor’s degree, master’s degree and above), religious beliefs (yes, no), family monthly income (<5000, 5000–10,000, >10,000 RMB), and employment status (unemployed, employed, student).

## 3. Questionnaires

### 3.1. Conspiracy Beliefs

“Conspiracy beliefs” was measured by two observed variables, including: (1) general coronavirus conspiracy beliefs, and (2) vaccine conspiracy beliefs. The coronavirus conspiracy scale of Coronavirus Explanations, Attitudes, and Narratives Survey (OCEANS) is a 7-item scale. Each item was rated on a 5-point scale ranging from 1 (do not agree) to 5 (agree completely), higher scores indicate higher conspiracy beliefs on general coronavirus [21], the Cronbach’s α was 0.787 in this study. The vaccine conspiracy beliefs scale is a 7-item scale, each item was rated on the 7-point Likert scale ranging from 1 (strongly disagree) to 7 (strongly agree), higher scores indicate higher endorsement of conspiracy beliefs on vaccines [28], the Cronbach’s α was 0.915 in this study. The model fit with the confirmatory factor analysis (CFA) was acceptable, indicating that “conspiracy beliefs” was a latent variable (supplementary materials, Tables S1 and S2). Endorsement of conspiracy beliefs is shown in the supplementary materials, Tables S11 and S12.

### 3.2. Medical Mistrust

Medical mistrust was measured by five observed variables including: (1) interpersonal disrespect by doctors, (2) negative views of vaccine developers, (3) negative attitude to doctors, (4) negative attitude to medicine, and (5) negative healthcare services experiences. The Oxford trust in doctors and developers questionnaire is a 16-item scale consisting of three subscales: (1) interpersonal disrespect by doctors; (2) respect from doctors; and (3) negative views of vaccine developers. Items were rated on a 4-point scale from 1 (disagree completely) to 4 (agree completely), higher scores indicate higher mistrust in doctors and vaccine developers [3], the Cronbach’s α was 0.951 in this study. The attitudes to doctors and medicine questionnaire is a 19-item scale, each item was rated on a 6-point scale from 1 (strongly disagree) to 6 (strongly agree), higher scores indicate higher mistrust in doctors and medicine [29], the Cronbach’s α was 0.808 in this study. The healthcare services experience questionnaire is an 8-item scale consisting of two subscales: positive and negative healthcare services experiences. Each item was rated on a 3-point scale ranging from 1 (No) to 3 (Yes), higher scores of negative subscales indicate higher mistrust in healthcare services experiences [3], the Cronbach’s α was 0.821 in this study. The model fit with the CFA was acceptable, indicating that medical mistrust was a latent variable (supplementary materials, Tables S3 and S4). Endorsement of mistrust is shown in the supplementary materials, Tables S13–S15.

### 3.3. Knowledge of Vaccines

Knowledge of vaccines was measured using the vaccination knowledge scale [30], a 9-item scale consisting of 2 subscales: (1) general knowledge about vaccines, and (2) knowledge about childhood vaccines. Each item was rated on a 0 (incorrect, or do not know) to 1 (correct) scale, higher scores indicate better knowledge of vaccines, the Cronbach’s α was 0.761 in this study. The model fit with the CFA was acceptable, indicating knowledge of vaccines was a latent variable (supplementary materials, Tables S7 and S8). Endorsement of knowledge of vaccines is shown in the supplementary materials, Table S16.

### 3.4. Vaccine Confidence and Complacency

Vaccine confidence and complacency was measured using the Oxford COVID-19 vaccine confidence and complacency Scale [3], a 14-item scale consisting of four subscales: (1) collective importance of COVID-19 vaccines, (2) speed of vaccine development, (3) efficacy of COVID-19 vaccines, and (4) side effects. Each item was rated from 1 to 5, with higher scores indicating higher negative vaccine confidence and complacency, the Cronbach’s α for the whole scale was 0.872, and for four subscales was 0.765, 0.775, 0.780, and 0.763 in this study. The model fit with the CFA was acceptable, indicating the vaccine confidence and complacency was a latent variable (supplementary materials, Tables S5 and S6). Endorsement of vaccine confidence and complacency is shown in the supplementary materials, Table S17.

### 3.5. Vaccine Hesitancy

Vaccine hesitancy was measured by the 7-item Oxford COVID-19 vaccine hesitancy scale [3], each item was rated on a 5-point scale ranging from 1 to 5, higher scores indicate higher vaccine hesitancy, the Cronbach’s α was 0.885 in this study. The model fit of the CFA indicated was acceptable (supplementary materials, Table S9). The endorsement of vaccine hesitancy statements is shown in supplementary materials, Table S10.

### 3.6. Statistical Analysis

The STATA 16.0 software (Stata Corporation, College Station, TX, USA) and AMOS 23.0 software (IBM Corporation, Armonk, NY, USA) were used to perform statistical analysis. Descriptive analysis was performed to evaluate the endorsement of conspiracy beliefs, medical mistrust, knowledge, confidence and complacency of vaccines, and vaccine hesitancy. The differences in vaccine hesitancy according to sociodemographic characteristics were analyzed by the Fisher’s Exact and Chi-squared tests. There were no missing values in this study.

The SEM approach was performed to analyze the direct and indirect pathways from conspiracy beliefs to vaccine hesitancy according to the conceptual framework, including two stages: (1) validation of the measurement model; and (2) fitting of the structural models. The measurement model was conducted by CFA to delineate the relationships among observed and latent variables, examine the interrelationships and covariation among observed variables and factor loadings, and ultimately the model fit indices were calculated [31]. In this study, CFA was used to evaluate the measurement models for conspiracy beliefs, medical mistrust, knowledge, confidence and complacency of vaccines, and vaccine hesitancy. The directionality of relationships among latent and observed variables were determined by the structural models. Multiple modification indices were conducted to achieve the acceptable model fit, including: χ^2^/df, values of less than 3, the root mean square error of approximate (RMSEA) and standardized root mean square residual (SRMR), values of less than 0.06, the comparative fit index (CFI) and incremental fit index (IFI), values of greater than 0.90 [32]. The bootstrap resampling procedures with 2000 samples and a bias-corrected 95% confidence interval (BC 95% CI) were used to examine the direct and indirect effects of each pathway for statistical significance. The total effects were calculated as the sum of the direct and indirect effects, mathematically expressed as: c = c′ + ab, where c = total effect, c′ = direct effect, ab = indirect effect [33]. A value of *p* < 0.05 indicated statistical significance.

### 3.7. Ethical Approval

Respondents were informed of the summary of program *purpose, procedures*, expected outcomes, benefits and risks of the study on the first page of the electronic questionnaires, and all respondents signed informed consent before completing the questionnaires. Respondents could withdraw from the study at any moment. The IP addresses were identified and duplicate respondents were eliminated, if respondents did not answer a question, the applet would not jump to the next question. A pilot study was conducted on 10 respondents to verify the validity, clarity, and consistency of questionnaires, which were not modified after the pilot study, the data were merged into the final sample. This study was conducted in accordance with the Declaration of Helsinki and approved by the Xuzhou Medical University Ethics Committee (ID number: XZ20210210).

## 4. Results

### 4.1. Sociodemographic Characteristics and the Distribution of Vaccine Hesitancy

The descriptive analysis and comparison by vaccine hesitancy of the sociodemographic characteristics are shown in Table 1. A total of 1015 respondents completed the questionnaires, 463 (45.6%) were male, 313 (30.8%) aged between 30–39 years, 634 (62.5%) lived in urban areas, 659 (64.9%) were married, 638 (62.9%) reported a bachelor’s degree, 61 (6.0%) reported having religious beliefs, 519 (51.1%) reported higher than RMB 10,000 family monthly income, and 520 (51.2%) were employed. Individuals aged between 30–39 years were more likely to be vaccine hesitant compared with individuals aged between 18–29 years and older than 40 years; individuals with higher education were more likely to be vaccine hesitancy than those with lower education (*p* < 0.01); individuals with lower family monthly income were more likely to be vaccine hesitancy than those with higher family monthly income (*p* < 0.01).

### 4.2. Correlations of Conspiracy Beliefs, Medical Mistrust, Knowledge of Vaccines, Vaccine Confidence and Complacency, and Vaccine Hesitancy

Table 2 revealed that the intercorrelations for all the observed and latent variables were statistically significant, which indicated that the SEM approach was appropriately conducted to examine the conceptual framework.

### 4.3. The Pathways from Conspiracy Beliefs to Vaccine Hesitancy

**Figure 2:**
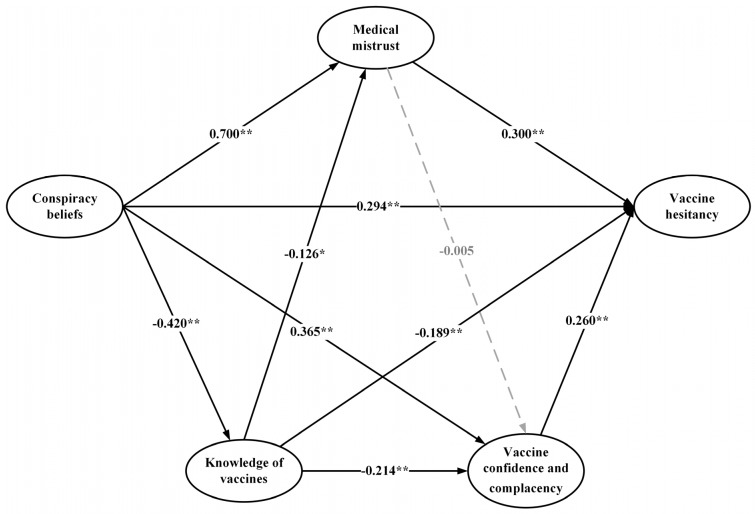
Standardized estimates of the pathways from conspiracy beliefs to Vaccine hesitancy of COVID-19 vaccines in the model. * *p* < 0.05, ** *p* < 0.01 Grey arrows refer to nonsignificant direct effects. Fitting of the model: χ^2^/df = 2.464; RMSEA = 0.038; SRMR = 0.050; CFI = 0.930; IFI = 0.930.

The standardized path estimates of the pathways from conspiracy beliefs to vaccine hesitancy are shown in Figure 2. The standardized estimates of the direct, indirect, and total effects of conspiracy beliefs on vaccine hesitancy as well as the specific effects through medical mistrust, knowledge, confidence and complacency of vaccines are presented in Table 3 and Table 4. Conspiracy beliefs had significant direct (*β* = 0.294), indirect (*β* = 0.423) and total (*β* = 0.717) effects on vaccine hesitancy, 41.0% of the total effect was direct, and 59.0% was indirect. Conspiracy beliefs significantly predicted vaccine hesitancy through medical mistrust (*β* = 0.210), knowledge (*β* = 0.079), confidence and complacency of vaccines (*β* = 0.095), explaining 29.3, 11.0, and 13.2% of the total effects, respectively. Conspiracy beliefs significantly predicted vaccine hesitancy through the sequential mediation of knowledge of vaccines and medical mistrust (*β* = 0.016), explaining 2.2% of the total effects. Conspiracy beliefs predicted vaccine hesitancy through the sequential mediation of knowledge of vaccines, and vaccine confidence and complacency (*β* = 0.023), explaining 3.2% of the total effects. The SEM approach indicated an acceptable model fit (χ^2^/df = 2.464, RMSEA = 0.038, SRMR = 0.050, CFI = 0.930, IFI = 0.930).

## 5. Discussion

This study contributes to the emerging picture of global COVID-19 vaccine hesitancy in the setting of LMICs through the identification of the pathways from conspiracy beliefs to vaccine hesitancy. The sample in this study showed lower vaccine hesitancy [7], with an 88.1% potential willingness to take COVID-19 vaccines without any hesitancy. However, due to the significant proportions of individuals either doubtful of, or rejecting of COVID-19 vaccines, 19.2% were likely to accept a COVID-19 vaccine, but 1.1% would “definitely not take” a COVID-19 vaccine. Conspiracy beliefs mainly had direct effect on vaccine hesitancy, and indirect association with vaccine hesitancy through medical mistrust, knowledge, confidence and complacency of vaccines, respectively. Conspiracy beliefs significantly indirectly predicted vaccine hesitancy through the sequential mediation of knowledge of vaccines, and medical mistrust; through the sequential mediation of knowledge of vaccines, and vaccine confidence and complacency. Both direct and indirect pathways from conspiracy beliefs to vaccine hesitancy were identified as intervention targets to reduce COVID-19 vaccine hesitancy.

With the implementation of vaccination programs, there has been a rise in vaccine hesitancy as a result of medical mistrust. Trust is an intrinsic and potentially modifiable component of the successful acceptance of the COVID-19 vaccines, which must be understood, and interventions crafted accordingly [7]. When individuals tend to medical mistrust, they are more likely to accept conspiracy beliefs, which produce short-term benefits, such as privileged information [10]. Strategies to trust and accept the COVID-19 vaccine are required if individuals are unwilling to be immunized and the uptake of a safe and effective COVID-19 vaccine is limited [7]. Trust in healthcare services is strongly associated with vaccine acceptance and can contribute to public compliance with recommended safety and prevention policies [7]. With negative perceptions for healthcare services, the danger is that the mistrust of COVID-19 vaccines may become mainstream [4]. Social and behavioural change strategies engaging local lower mistrust in healthcare workers may be effective in combating vaccine hesitancy [34]. Engaging trusted healthcare services should work to satisfy local needs and preferences, policymakers should expand access by offering flexible times, access sites, and various administering for COVID-19 vaccines [35]. Healthcare workers delivering the trusted information to guide COVID-19 vaccination, highlighting vaccine efficacy and safety, may be effective in reducing the remaining hesitancy [15]. Receiving the COVID-19 vaccines from trusted healthcare workers is imperative to identify targeted strategies to educate and advise vaccine hesitant individuals [26]. Conspiracy beliefs foster medical mistrust and erode social cohesion to decrease vaccine uptake, indicates that prosocial benefits may be effective for the COVID-19 vaccines [3], which may increase the willingness of hesitant individuals to consolidate vaccination uptake [20].

The most commonly reported reason for vaccine hesitancy is concerned about side effects, which may due to the rapid development of the vaccines [36]. Side effects associated with vaccine development may increase vaccine hesitancy, and the COVID-19 fast-moving pandemic and vaccine development may change perceptions about the vaccine [15]. Vaccine hesitancy was around side effects for intended vaccine uptake related to the avoidance of the COVID-19, and knowledge of vaccine efficacy and safety was found to encourage vaccine uptake in the UK [37]. There is a substantial view among the Chinese public that COVID-19 vaccines are safe and effective, but policymakers should provide knowledge of COVID-19 vaccine, especially for individuals rejecting it to effectively control the COVID-19 pandemic [38]. There is a necessity to be transparent about safety and efficacy [20], clear and consistent communication polices to explain how vaccines work, developed and approval based on safety and efficacy, are crucial to build public confidence in the COVID-19 vaccine [7]. 

The COVID-19 pandemic and associated policies set the conditions for the development of conspiracy beliefs [7]. Vaccine hesitancy is strongly bound to vaccine conspiracy beliefs, which presents a great challenge for government agencies that need to combat misinformation to ensure the uptake of COVID-19 vaccines [14]. The COVID-19 vaccines are being offered to a public that is suffering from pandemic fatigue, while misinformation related to conspiracy beliefs is perpetuated [38]. There is an urgent need to counter vaccine misinformation, and provide accurate presentation; the long-term task is to build trust in healthcare services [20]. Written information combined with images and videos may be beneficial when the information is reinforced by trusted acquaintances or healthcare workers through face-to-face, or social media apps [4]. The social media platform promotes the dissemination of valid information, and provides interventions to limit the circulation of misinformation [14]. Developing vaccine acceptance strategies should directly consider misconceptions and skepticism and be sensitive to philosophical beliefs [20]. Policymakers must identify trusted local sources of information, provide initial guidance, and state that vaccines are safe, effective, and essential to control COVID-19 [39]. 

Willingness to be vaccinated is influenced by factors, such as general health knowledge, whether vaccine uptake is perceived to be effective, and concerns about side effects [40], which may be the key drivers for vaccine hesitancy [37]. The behaviour of individuals who are ambivalent about vaccinations may be a determining factor in the success of implementation of vaccination campaigns [10]. Multiple strategies, such as increasing knowledge and access to vaccines, are effective in addressing vaccine hesitancy [28]; individuals with the vaccine relevant knowledge in LMICs have been shown to prefer to follow the COVID-19 prevention guidelines [15]. Brief vaccine information provided with the statement of efficacy and safety may change vaccine hesitancy in those who are not willing to be vaccinated or doubtful [4]. Communicating accurate information about potential side effects may contribute to a decrease in vaccine hesitancy through social media coverage [11]. Policymakers should consider designing and evaluating social mobilization strategies targeted at hesitant individuals [41]; social learning and mobilization strategies are valuable for positive attitudes towards COVID-19 vaccines, which may shift social norms toward greater vaccine acceptance and uptake [42]. The COVID-19 vaccines must be widely accepted by the public and healthcare services to confer population benefit [43], proactive messaging should be initiated before large-scale vaccination campaign rollout, policymakers should highlight the efficacy and safety of vaccines in controlling COVID-19, reducing hospitalizations and death. Guidance on the specific content of vaccine is likely to be most effective in persuading hesitant individuals who are concerned about side effects and efficacy of vaccines; a balance between educating the public about the necessary coverage for COVID-19 vaccine and avoiding coercion policies is needed [7]. 

Policymakers should identify the pathways between conspiracy beliefs and vaccine hesitancy for a safe and successful COVID-19 vaccination campaign [7]. COVID-19 vaccine hesitancy is spread across the population, and willingness to accept the vaccine is strongly linked to recognition of the collective importance, which is critical to achieving high coverage [3]. Multiple features of the COVID-19 pandemic link to the development and propagation of conspiracy beliefs, and individual beliefs may affect the adherence to collective importance, erroneous beliefs foster useless behaviours [10], which weaken collective actions to minimize harm to the individuals [7]. Individuals with vaccination hesitancy lack confidence and complacency as well as public willingness to accept the COVID-19 vaccine, and the assessment of vaccination confidence and complacency reveals a decline in acceptance of vaccines with increasing vaccine hesitancy [38]. Currently, there may be the largest vaccine campaign in the history of the world, and yet a decline in public confidence in vaccination incentive policies, such as rewarding those who receive the vaccine. Due to concerns about the efficacy and safety of the vaccine, there is a decline in public confidence in vaccination, the ongoing COVID-19 pandemic revealing that it is crucial to build vaccine confidence to support vaccine uptake [7], which is influenced by trust in the safety and effectiveness of vaccines, healthcare workers, healthcare services, and policymakers [44]. While universal and targeted vaccine interventions are needed to enable individuals to understand the importance of vaccination, such as modifying behaviour or increasing confidence [45], full endorsement from regulatory bodies is likely to increase confidence, but efforts to combat mistrust of vaccine safety may be necessary [46]. Policymakers should focus on vaccine hesitant individuals in national polls, and the persuasive power of access to trusted healthcare workers embedded in the communities.

Vaccine hesitancy compromises the success of COVID-19 vaccination campaigns, but securing adequate acceptance of vaccines may decrease vaccine hesitancy. How quickly policymakers can convince individuals to be vaccinated would have an impact on the final death toll due to COVID-19, leveraging early acceptors to overcome vaccine hesitancy [47]. This study suggests several concrete implications relating to vaccine rollout in LMICs, and directions for the design and delivery of information to decrease COVID-19 vaccine hesitancy. In times of the COVID-19 pandemic, integrating rigorous evaluation of vaccine hesitancy interventions in all contexts is imperative [15]. Policymakers and stakeholders should use country-specific strategies that may work best in particular contexts, assessing the local context, understanding relevant barriers and resources, and assisting the efficient adoption of the COVID-19 vaccine interventions [43]. The government agencies are required to select evidence-based strategies to identify interventions that are acceptable and feasible, to increase COVID-19 vaccine uptake, tailored to the specific needs and resources [48]. Promising intervention policies for reducing vaccine hesitancy should be considered in different contexts, particularly, awareness and attention to existing public perceptions [12]. It is a multifactorial, complex and context-dependent endeavor to reduce COVID-19 vaccine hesitancy that requires more than building trust [7]; the success of a vaccination campaign is contingent upon scientific data combined with higher acceptance and coverage [43]. Implementation of evidence-based strategies at the international, organizational, and interpersonal levels to reduce vaccine hesitancy is imperative to successfully achieve the vaccination levels of the population necessary to end the COVID-19 pandemic [7]. 

## 6. Strength and Limitations

This study has both strengths and limitations. To our knowledge, it is the first study in LMICs to explore the pathways from conspiracy beliefs to vaccine hesitancy; the findings reveal that the identified direct and indirect pathways from conspiracy beliefs to vaccine hesitancy can be intervention targets to reduce COVID-19 vaccine hesitancy. This study was a snapshot taken at one point in time, and COVID-19 is a highly dynamic and changing landscape, with daily variations in perceived COVID-19 threat and vaccine development itself. We caution that this study was a non-probability survey, and while better than a purposive sample, it still has introduced bias. It was not determined whether the findings can be generalized to China or other countries. Further study needs to enrich the large representative participants in longitudinal study for COVID-19 vaccine hesitancy worldwide.

## 7. Conclusions

Permanent refusal of all vaccines should be rare, as individuals who initially refuse vaccines may eventually change their behaviours [47]. This study identified the pathways from conspiracy beliefs to COVID-19 vaccine hesitancy in China. Conspiracy beliefs had mainly direct effects on vaccine hesitancy, and indirect effects on vaccine hesitancy through medical mistrust, knowledge, confidence and complacency of vaccines. The recommendation should be consistent with the accepted framework to reduce COVID-19 vaccine hesitancy. This study may help government agencies, policymakers, and healthcare workers to target effective vaccine hesitancy intervention programs around COVID-19 vaccination, and longitudinal monitoring of vaccine hesitancy is required.

## Figures and Tables

**Table 1:** Descriptive statistics of sociodemographic characteristics (*N* = 1015).

Variables	Vaccine Hesitancy					
	*n* (%)	Willing (*n* = 894)	Doubtful (*n* = 82)	Strongly Hesitancy (*n* = 39)	χ^2^	*p*
Sex					7.407	0.023
Male	463 (45.6)	419 (90.5)	34 (7.3)	10 (2.2)		
Female	552 (54.4)	475 (86.1)	48 (8.7)	29 (5.3)		
Age					13.097	0.041
18–29 years	275 (27.1)	254 (92.4)	14 (5.1)	7 (2.5)		
30–39 years	313 (30.8)	260 (83.1)	36 (11.5)	17 (5.4)		
40–49 years	290 (28.6)	256 (88.3)	23 (7.9)	11 (3.8)		
≥50 years	137 (13.5)	124 (90.5)	9 (6.6)	4 (2.9)		
Area of residence					0.354	0.838
Rural	381 (37.5)	338 (88.7)	30 (7.9)	13 (3.4)		
Urban	634 (62.5)	556 (87.7)	52 (8.2)	26 (4.1)		
Marital status					3.288	0.463
Single	333 (32.8)	289 (86.8)	31 (9.3)	13 (3.9)		
Married	659 (64.9)	586 (88.9)	49 (7.4)	24 (3.6)		
Others	23 (2.3)	19 (82.6)	2 (8.7)	2 (8.7)		
Educational level					13.319	0.031
Middle school and below	55 (5.4)	52 (94.5)	2 (3.6)	1 (1.8)		
Senior high school	92 (9.1)	87 (94.6)	3 (3.3)	2 (2.2)		
Bachelor’s degree	638 (62.9)	565 (88.6)	53 (8.3)	20 (3.1)		
Master’s degree and above	230 (22.7)	190 (82.6)	24 (10.4)	16 (7.0)		
Religious beliefs					1.193	0.522
Yes	61 (6.0)	52 (85.2)	7 (11.5)	2 (3.3)		
No	954 (94.0)	842 (88.3)	75 (7.9)	37 (3.9)		
Family monthly income (RMB)					29.703	< 0.001
<5000	96 (9.5)	68 (70.8)	23 (24.0)	5 (5.2)		
5000–10,000	400 (39.4)	364 (91.0)	25 (6.3)	11 (2.8)		
>10,000	519 (51.1)	462 (89.0)	34 (6.6)	23 (4.4)		
Employment status					0.778	0.943
Unemployed	168 (16.6)	146 (86.9)	15 (8.9)	7 (4.2)		
Employed	520 (51.2)	456 (87.7)	43 (8.3)	21 (4.0)		
Student	327 (32.2)	292 (89.3)	24 (7.3)	11 (3.4)		

**Table 2:** Correlation matrix of the variables (*N* = 1015).

Variables	(1)	(2)	(3)	(4)	(5)	(6)	(7)	(8)	(9)	(10)	(11)	(12)	(13)	(14)	(15)	(16)	(17)	(18)
**Observed variables**																		
General coronavirus conspiracy beliefs (1)	1																	
Vaccine conspiracy beliefs (2)	0.59 *	1																
Disrespect from doctors (3)	0.43 *	0.60 *	1															
Negative views of vaccine developers (4)	0.44 *	0.61 *	0.80 *	1														
Negative attitude to doctors (5)	0.45 *	0.62 *	0.82 *	0.83 *	1													
Negative attitude to medicine (6)	0.37 *	0.51 *	0.67 *	0.68 *	0.70 *	1												
Negative health care service experiences (7)	0.34 *	0.47 *	0.61 *	0.62 *	0.64 *	0.52 *	1											
Collective importance (8)	0.29 *	0.40 *	0.32 *	0.32 *	0.33 *	0.27 *	0.25 *	1										
Speed of vaccine development (9)	0.23 *	0.32 *	0.25 *	0.25 *	0.26 *	0.21 *	0.19 *	0.77 *	1									
Efficacy of COVID-19 vaccines (10)	0.28 *	0.38 *	0.30 *	0.30 *	0.31 *	0.26 *	0.23 *	0.93 *	0.73 *	1								
Side effects (11)	0.25 *	0.35 *	0.27 *	0.28 *	0.28 *	0.23 *	0.21 *	0.85 *	0.67 *	0.80 *	1							
General knowledge about vaccines (12)	−0.28 *	−0.39 *	−0.38 *	−0.39 *	−0.40 *	−0.33 *	−0.30 *	−0.37 *	−0.29 *	−0.35 *	−0.32 *	1						
Knowledge about childhood vaccines (13)	−0.29 *	−0.40 *	−0.39 *	−0.40 *	−0.41 *	−0.33 *	−0.30 *	−0.38 *	−0.30 *	−0.36 *	−0.33 *	0.96 *	1					
**Latent variables**																		
Conspiracy beliefs (14)	0.65 *	0.90 *	0.67 *	0.68 *	0.69 *	0.57 *	0.52 *	0.45 *	0.35 *	0.42 *	0.39 *	−0.43 *	−0.44 *	1				
Knowledge of vaccines (15)	−0.27 *	−0.38 *	−0.37 *	−0.38 *	−0.39 *	−0.32 *	−0.29 *	−0.36 *	−0.29 *	−0.34 *	−0.31 *	0.99 *	0.97 *	−0.42 *	1			
Medical mistrust (16)	0.49 *	0.68 *	0.88 *	0.90 *	0.92 *	0.76 *	0.69 *	0.36 *	0.28 *	0.34 *	0.31 *	−0.43 *	−0.44 *	0.75 *	−0.42 *	1		
Vaccine confidence and complacency (17)	0.29 *	0.41 *	0.32 *	0.32 *	0.33 *	0.27 *	0.25 *	0.99 *	0.78 *	0.94 *	0.85 *	−0.37 *	−0.38 *	0.45 *	−0.37 *	0.36 *	1	
Vaccine hesitancy (18)	0.47 *	0.64 *	0.61 *	0.63 *	0.64 *	0.53 *	0.48 *	0.57 *	0.45 *	0.53 *	0.49 *	−0.55 *	−0.56 *	0.72 *	−0.53 *	0.70 *	0.57 *	1

* *p* < 0.01.

**Table 3:** Standardized indirect path effects from conspiracy beliefs to vaccine hesitancy (*N* = 1015).

Pathways	Effect	S.E.	*p*	BC 95% CI
Conspiracy beliefs → Medical mistrust → Vaccine hesitancy	0.210	0.048	0.001	0.106 to 0.301
Conspiracy beliefs → Vaccine confidence and complacency → Vaccine hesitancy	0.095	0.034	0.001	0.027 to 0.100
Conspiracy beliefs → Knowledge of vaccines → Vaccine hesitancy	0.079	0.022	0.001	0.029 to 0.163
Conspiracy beliefs → Mistrust → Vaccine confidence and complacency → Vaccine hesitancy	−0.001	0.020	0.987	0.038 to 0.987
Conspiracy beliefs → Knowledge of vaccines → Medical mistrust → Vaccine hesitancy	0.016	0.008	0.015	0.032 to 0.015
Conspiracy beliefs → Knowledge of vaccines → Vaccine confidence and complacency → Vaccine hesitancy	0.023	0.010	0.003	0.045 to 0.003
Conspiracy beliefs → Knowledge of vaccines → Medical mistrust → Vaccine confidence and complacency → Vaccine hesitancy	<0.001	0.002	0.994	−0.003 to 0.003

S.E.: standard error; BC 95% CI: bias-corrected 95% confidence intervals.

**Table 4:** Standardized direct effect, indirect and total effect of the variables on vaccine hesitancy (*N* = 1015).

Variables	Standardized Direct Effects	Standardized Indirect Effects	Standardized Total Effects
Conspiracy beliefs	0.294 *	0.423 *	0.717 *
Medical mistrust	0.300 *	−0.001	0.299 *
Knowledge of vaccines	−0.189 *	−0.093 *	−0.282 *
Vaccine confidence and complacency	0.260 *	-	0.260 *

* *p* < 0.01.

## Data Availability

The datasets generated and analysed during the current study are not publicly available due to original consent, but are available from the corresponding author upon reasonable request.

## Supplementary Materials

The following are available online at https://www.mdpi.com/article/10.3390/vaccines9111342/s1, Table S1: Factor correlations of CFA model, Table S2: Standardized factor loadings of CFA model with Conspiracy beliefs, Table S3: Factor correlations of CFA model, Table S4: Standardized factor loadings of CFA model with Medical mistrust, Table S5: Factor correlations of CFA model, Table S6: Standardized factor loadings of CFA model with Knowledge of vaccines, Table S7: Factor correlations of CFA model, Table S8: Standardized factor loadings of CFA model with Vaccine confidence and complacency, Table S9: Standardized loadings based on confirmatory factor loadings, Table S10: Endorsement of vaccine hesitancy items, Table S11: Endorsement of general coronavirus conspiracy beliefs items, Table S12: Endorsement of vaccine conspiracy beliefs items, Table S13: Endorsement of Oxford trust in doctors and developers items, Table S14: Endorsement of attitudes to doctors and medicine items, Table S15: Endorsement of negative healthcare services experiences items, Table S16: Endorsement of knowledge of vaccines items, Table S17: Endorsement of vaccine confidence and complacency items.
