# Supplementary material for: The Mediating Roles of Medical Mistrust, Knowledge, Confidence and Complacency of Vaccines in the Pathways from Conspiracy Beliefs to Vaccine Hesitancy
Source: Vaccines (Basel). 2021 Nov 17;9(11):1342. doi: 10.3390/vaccines9111342 (PMC8624917; doi:10.3390/vaccines9111342)
Supplement: Supplementary file 1 [file vaccines-09-01342-s001.zip › vaccines-1416645-supplementary.pdf]

Supplementary materials: results of CFA and Questionnaires items

Conspiracy beliefs

Initial Confirmatory factor analysis (CFA) showed that item A2 had loadings of less than 0.4 and was therefore, was removed from analysis. The final model indicated a good fit (RMSEA = 0.039; SRMR = 0.024; CFI = 0.984; GFI = 0.976).

Table S1. Factor correlations of CFA model

Constructs	(1)	(2)
General coronavirus conspiracy beliefs (1)	1.00	
Conspiracy beliefs on vaccines (2)	0.91*	1.00

*$p < 0.01$.

Table S2. Standardized factor loadings of CFA model with Conspiracy beliefs

Items	Factors	Factor loadings	Mean	SD
A1	General coronavirus conspiracy beliefs	0.61	1.22	0.59
A3		0.57	1.36	0.78
A4		0.62	1.32	0.74
A5		0.62	1.36	0.76
A6		0.62	1.35	0.77
A7		0.67	1.35	0.73
B1	Conspiracy beliefs on vaccines	0.81	2.27	1.51
B2		0.81	2.29	1.45
B3		0.76	2.41	1.56
B4		0.86	2.37	1.52
B5		0.66	2.15	1.39
B6		0.77	2.27	1.53
B7		0.77	2.24	1.47
General coronavirus conspiracy beliefs	Conspiracy beliefs	0.91	9.27	3.52
Conspiracy beliefs on vaccines		0.65	16.00	8.30

SD: Standard deviation.

Medical mistrust

Initial CFA showed that item E2 and item F1 had loadings of less than 0.4 and were therefore, were removed from analysis. The final model indicated a good fit (RMSEA = 0.057; SRMR = 0.024; CFI = 0.970; GFI = 0.937).

Table S3. Factor correlations of CFA model

Constructs	(1)	(2)	(3)	(4)	(5)
Disrespect from doctors (1)	1.00				
Negative views of vaccine developers (2)	0.82*	1.00			
Negative attitude to doctors (3)	0.79*	0.83*	1.00		
Negative attitude to medicine (4)	0.64*	0.67*	0.80*	1.00	
Negative health care services experiences (5)	0.56*	0.58*	0.70*	0.56*	1.00

*$p < 0.01$.

Table S4. Standardized factor loadings of CFA model with Medical mistrust

Items	Factors	Factor Loadings	Mean	SD
C1	Disrespect from doctors	0.86	1.69	0.76
C2		0.88	1.72	0.74
C3		0.69	2.04	0.81
C4		0.89	1.80	0.71
C5		0.93	1.73	0.72
C6		0.93	1.71	0.74
D1	Negative views of vaccine developers	0.90	1.80	0.78
D2		0.92	1.75	0.75
D3		0.93	1.78	0.75
D4		0.91	1.77	0.75
E1	Negative attitude to doctors	0.76	2.26	1.29
E3		0.87	2.26	1.21
E4		0.89	2.11	1.18
F2	Negative attitude to medicine	0.63	3.05	1.42
F3		0.86	2.49	1.32
F4		0.80	2.56	1.38
G1	Negative health care services experiences	0.62	1.68	0.75
G2		0.78	1.51	0.70
G3		0.77	1.69	0.76
G4		0.78	1.72	0.74
Disrespect from doctors	Medical mistrust	0.80	10.69	3.97
Negative views of vaccine developers		0.83	7.10	2.82
Negative attitude to doctors		0.94	6.62	3.28

Negative attitude to medicine	0.80	8.10	3.50
Negative health care services experiences	0.70	6.14	2.24

SD: Standard deviation.

Knowledge of vaccines

Initial CFA showed that item H2 had loadings of less than 0.4 and was therefore, was removed from analysis. The final model indicated a good fit (RMSEA = 0.049; SRMR = 0.034; CFI = 0.946; GFI = 0.988).

Table S5. Factor correlations of CFA model

Constructs	(1)	(2)
General Knowledge about vaccines (1)	1.00	
Knowledge about childhood vaccines (2)	0.85*	1.00

*$p < 0.01$.

Table S6. Standardized factor loadings of CFA model with Knowledge of vaccines

Items	Factors	Factor loadings
H1	General Knowledge about vaccines	0.67
H3		0.65
H4		0.68
H5	Knowledge about childhood vaccines	0.58
H6		0.68
H8		0.72
H9		0.69
General Knowledge about vaccines	Knowledge of vaccines	0.68
Knowledge about childhood vaccines		0.71

Vaccine confidence and complacency

Initial CFA showed that item J1 had loadings of less than 0.4 and was therefore, was removed from analysis. The final model indicated a good fit (RMSEA = 0.049; SRMR = 0.034; CFI = 0.946; GFI = 0.988).

Table S7. Factor correlations of CFA model

Constructs	(1)	(2)	(3)	(4)
Vaccine will be effective (1)	1.00			
Speed of vaccine development (2)	0.72*	1.00		
Collective importance (3)	0.92*	0.74*	1.00	
Side effects (4)	0.82*	0.66*	0.84*	1.00

*p < 0.01.

Table S8. Standardized factor loadings of CFA model with Vaccine confidence and complacency

Items	Factors	Factor loadings	Mean	SD
J2	Efficacy of COVID-19 vaccines	0.69	2.01	0.59
J3		0.74	1.88	0.86
J4	Speed of vaccine development	0.91	1.93	0.93
J5		0.85	2.03	0.92
J6		0.82	2.14	0.83
J7	Collective importance	0.81	1.56	0.80
J8		0.75	1.40	0.75
J9		0.58	1.88	0.71
J10		0.59	2.34	0.82
J11		0.56	2.23	0.92
J12	Side effects	0.63	1.96	0.70
J13		0.74	1.87	0.71
J14		0.55	2.01	1.01
Efficacy of COVID-19 vaccines	Vaccine confidence and complacency	0.95	3.89	1.27
Speed of vaccine development		0.76	6.10	2.24
Collective importance		0.97	9.40	2.93
Side effects		0.87	5.84	1.87

SD: Standard deviation.

Vaccine hesitancy

CFA model indicated a good fit (RMSEA = 0.056; SRMR = 0.020; CFI = 0.988; GFI = 0.983).

Table S9. Standardized loadings based on confirmatory factor loadings

Item	Factor loading	Mean	SD
K1	0.76	1.45	0.81
K2	0.75	1.52	0.90
K3	0.81	1.73	0.85
K4	0.82	1.65	0.83
K5	0.79	1.73	0.81
K6	0.76	1.83	0.72
K7	0.42	1.54	0.89

Standardized item loadings for a single factor, SD: Standard deviation.

Table S10. Endorsement of vaccine hesitancy items

Items	Response	*n*	%
K1. Would you take a COVID-19 vaccine (approved for use in China) if offered?	Definitely	712	70.1
	Probably	195	19.2
	I may or I may not	76	7.5
	Probably not	21	2.1
	Definitely not	11	1.1
K2. If there is a COVID-19 vaccine available,	I will want to get it as soon as possible	702	69.2
	I will take it when offered	157	15.5
	I'm not sure what I will do	117	11.5
	I will put off (delay) getting it	23	2.3
	I will refuse to get it	16	1.6
K3. I would describe my attitude towards receiving a COVID-19 vaccine as:	Very keen	477	47.0
	Pretty positive	381	37.5
	Neutral	123	12.1
	Quite uneasy	21	2.1
	Against it	13	1.3
K4. If a COVID-19 vaccine was available at my local pharmacy, I would:	Get it as soon as possible	532	52.4
	Get it when I have time	353	34.8
	Delay getting it	94	9.3

	Avoid getting it for as long as possible	26	2.6
	Never get it	10	1.0
K5. If my family or friends were thinking of getting a COVID-19 vaccination, I would:	Strongly encourage them	428	42.2
	Encourage them	460	45.3
	Not say anything to them about it	92	9.1
	Ask them to delay getting the vaccination	22	2.2
	Suggest that they do not get the vaccination	13	1.3
K6. I would describe myself as:	Eager to get a COVID-19 vaccine	309	30.4
	Willing to get the COVID-19 vaccine	607	59.8
	Not bothered about getting the COVID-19 vaccine	71	7.0
	Unwilling to get the COVID-19 vaccine	17	1.7
	Anti-vaccination for COVID-19	11	1.1
K7. Taking a COVID-19 vaccination is:	Really important	540	53.2
	Important	408	40.2
	Neither important nor unimportant	46	4.5
	Unimportant	13	1.3
	Really unimportant	8	0.8

Table S11. Endorsement of general coronavirus conspiracy beliefs items

Items	Do not agree	Agree a little	Agree moderately	Agree a lot	Agree completely
A1. The virus is a hoax.	867 (85.4%)	92 (9.1%)	43 (4.2%)	11 (1.1%)	2 (0.2%)
A2. The virus is manmade.	870 (85.7%)	46 (4.5%)	51 (5.0%)	22 (2.2%)	26 (2.6%)
A3. The spread of the virus is a deliberate attempt to reduce the size of the global population.	780 (76.8%)	150 (14.8%)	47 (4.6%)	29 (2.9%)	9 (0.9%)
A4. The spread of the virus is a deliberate attempt by governments to gain political control.	804 (79.2%)	136 (13.4%)	45 (4.4%)	20 (2.0%)	10 (1.0%)
A5. The spread of the virus is a deliberate attempt by a group of powerful people to make money.	781 (76.9%)	147 (13.8%)	52 (5.1%)	28 (2.8%)	7 (0.7%)
A6. The spread of the virus is a deliberate attempt by one nation to destabilize another.	787 (77.5%)	140 (13.8%)	52 (5.1%)	28 (2.8%)	8 (0.8%)
A7. The spread of the virus is a deliberate attempt by global companies to take control.	779 (76.7%)	149 (14.7%)	58 (5.7%)	25 (2.5%)	4 (0.4%)

Table S12. Endorsement of vaccine conspiracy beliefs items

Items	Strongly disagree	Disagree	Somewhat disagree	Neutral	Somewhat agree	Agree	Strongly agree
B1. Vaccine safety data are often fabricated (made up).	361 (35.6%)	392 (38.6%)	95 (9.4%)	56 (5.5%)	46 (4.5%)	38 (3.7%)	27 (2.7%)
B2. Immunizing children is harmful and this fact is covered up.	329 (32.4%)	411 (40.5%)	105 (10.3%)	65 (6.4%)	46 (4.5%)	40 (3.9%)	19 (1.9%)
B3. Pharmaceutical companies cover up the dangers of vaccines.	327 (32.2%)	375 (36.9%)	119 (11.7%)	63 (6.2%)	56 (5.5%)	49 (4.8%)	26 (2.6%)
B4. People are deceived about the effectiveness of vaccines.	310 (30.5%)	417 (41.1%)	109 (10.7%)	57 (5.6%)	47 (4.6%)	50 (4.9%)	25 (2.5%)
B5. Vaccine effectiveness data are often fabricated (made up).	346 (34.1%)	474 (46.7%)	62 (6.1%)	35 (3.4%)	43 (4.2%)	38 (3.7%)	17 (1.7%)
B6. People are deceived about vaccine safety.	394 (38.8%)	322 (31.7%)	138 (13.6%)	51 (5.0%)	47 (4.6%)	33 (3.3%)	30 (3.0%)
B7. The government is trying to cover up the link between vaccines and autism.	382 (37.6%)	343 (33.8%)	132 (13.0%)	59 (5.8%)	40 (3.9%)	37 (3.6%)	22 (2.2%)

Table S13. Endorsement of Oxford trust in doctors and developers items

Items	Disagree completely	Disagree	Agree	Agree completely
C1. They do not really care about me.	464 (45.7%)	428 (42.2%)	93 (9.2%)	30 (3.0%)
C2. They have little respect for me.	437 (43.1%)	456 (44.9%)	96 (9.5%)	26 (2.6%)
C3. They have no idea what my life is like.	282 (27.8%)	438 (43.2%)	266 (26.2%)	29 (2.9%)
C4. They often make mistakes.	356 (35.1%)	537 (52.9%)	96 (9.5%)	26 (2.6%)
C5. They are in it for the money.	409 (40.3%)	493 (48.6%)	86 (8.5%)	27 (2.7%)
C6. I do not trust doctors.	441 (43.4%)	455 (44.8%)	92 (9.1%)	27 (2.7%)
D1. I do not trust the vaccine developers.	389 (38.3%)	477 (47.0%)	110 (10.8%)	39 (3.8%)
D2. They just want to make money.	409 (40.3%)	479 (47.2%)	97 (9.6%)	30 (3.0%)
D3. They do not care about helping people.	389 (38.3%)	490 (48.3%)	106 (10.4%)	30 (3.0%)
D4. They do not properly check that the vaccine is safe.	395 (38.9%)	491 (48.4%)	98 (9.7%)	31 (3.1%)

Table S14. Endorsement of attitudes to doctors and medicine items

Items	Strongly disagree	Disagree	Tend to disagree	Tend to agree	Agree	Strongly agree
E1. Doctors blame their patients if their treatment doesn't work.	311 (30.6%)	412 (40.6%)	118 (11.6%)	101 (10.0%)	37 (3.6%)	36 (3.5%)
E2. No two doctors will agree with what is wrong with someone.	167 (16.5%)	404 (39.8%)	234 (23.1%)	76 (7.5%)	78 (7.7%)	56 (5.5%)
E3. Doctors are too ready to solve patients' problems by prescribing tranquillizers.	293 (28.9%)	408 (40.2%)	156 (15.4%)	105 (10.3%)	24 (2.4%)	29 (2.9%)
E4. I don't like medical people.	350 (34.5%)	414 (40.8%)	118 (11.6%)	85 (8.4%)	23 (2.3%)	25 (2.5%)
F1. Medicines can do as much harm as good.	226 (26.2%)	463 (45.6%)	151 (14.9%)	47 (4.6%)	35 (3.4%)	53 (5.2%)
F2. Many medicines are just placebos or sugar pills.	146 (14.4%)	280 (27.6%)	193 (19.0%)	224 (22.1%)	118 (11.6%)	54 (5.3%)
F3. Often the only purpose of tests is to make a doctor feel less anxious.	240 (23.6%)	383 (37.7%)	176 (17.3%)	124 (12.2%)	52 (5.1%)	40 (3.9%)
F4. Most tests and investigations are done routinely rather than for a particular purpose.	243 (23.9%)	364 (35.9%)	159 (15.7%)	142 (14.0%)	62 (6.1%)	45 (4.4%)

Table S15. Endorsement of negative healthcare services experiences items

Items	No	Maybe	Yes
G1. Staff have gone out of their way to help.	498 (49.1%)	344 (33.9%)	173 (17.0%)
G2. Received harmful care.	615 (60.6%)	280 (27.6%)	120 (11.8%)
G3. Family received harmful care.	615 (60.6%)	297 (29.3%)	103 (10.1%)
G4. The NHS treats your community badly.	641 (63.2%)	284 (28.0%)	90 (8.9%)

Table S16. Endorsement of knowledge of vaccines items

Items	Incorrect	Correct
H1. Vaccines are superfluous (unnecessary), as diseases can be treated (e.g., with antibiotics).	885 (87.2%)	130 (12.8%)
H2. Without broadly applied vaccine programmers, smallpox would still exist.	191 (18.8%)	824 (81.2%)
H3. The efficacy of vaccines has been proven.	184 (18.1%)	831 (81.9%)
H4. The doses of the vaccines are not dangerous for humans.	221 (21.8%)	794 (78.2%)
H5. Children would be more resistant if they were not always vaccinated against all diseases.	834 (82.2%)	181 (17.8%)
H6. Diseases like autism, multiple sclerosis, and diabetes might be triggered through vaccination.	188 (18.5%)	827 (81.5%)
H7. The immune system of children is not overloaded through many vaccinations.	529 (52.1%)	486 (47.9%)
H8. Many vaccinations are administered too early, so that the body's own immune system has no possibility to develop.	694 (68.4%)	321 (31.6%)
H9. Vaccinations increase the occurrence of allergies.	665 (65.5%)	350 (34.5%)

Table S17. Endorsement of vaccine confidence and complacency items

Items	Response	*n*	%
J1. Do you think you will be infected with COVID-19 over the next 12 months?	Definitely	529	52.1
	Probably	269	26.5
	Possibly	133	13.1
	Probably not	47	4.6
	Definitely not	37	3.6
J2. The COVID-19 vaccine is likely to:	Work for almost everyone	136	13.4
	Work for most people	762	75.1
	I am unsure how many people it will work for	94	9.3
	Not work for most people	17	1.7
	Not work for anyone	6	0.6
J3. The COVID-19 vaccine is likely to:	Definitely work for me	362	35.7
	Probably work for me	478	47.1
	May or may not work for me	129	12.7
	Probably not work for me	28	2.8
	Definitely not work for me	18	1.8
J4. The speed of developing and testing the vaccine means it will be:	Really good	386	38.0
	Good	383	37.7
	Will not affect how good or bad it is	195	19.2

	Bad	34	3.3
	Really bad	17	1.7
J5. The speed of developing and testing the vaccine means it will be:	Really safe	322	31.7
	Safe	419	41.3
	It will not affect how safe it is	210	20.7
	Unsafe	50	4.9
	Really unsafe	14	1.4
J6. Getting the vaccine is a sign of:	Great personal strength	237	23.3
	Personal strength	436	43.0
	Not a sign of personal strength or weakness	315	31.0
	Personal weakness	16	1.6
	Great personal weakness	11	1.1
J7. If I get the COVID-19 vaccine it will be:	Really helpful for the community around me	578	56.9
	Helpful for the community around me	360	35.5
	Neither helpful nor unhelpful for the community around me	43	4.2
	Unhelpful for the community around me	16	1.6
	Really unhelpful for the community around me	18	1.8
J8. If individuals like me get the COVID-19 vaccine it will:	Save a large number of lives	717	70.6
	Save some lives	234	23.1
	Have no impact	27	2.7

	Lead to more deaths	28	2.8
	Lead to a large number of deaths	9	0.9
J9. If many people do not get the vaccine this:	Will be dangerous	264	26.0
	May be dangerous	656	64.6
	Will have no consequences at all	61	6.0
	May be good	21	2.1
	Will be good	13	1.3
J10. The COVID-19 vaccine will:	Greatly strengthen my immune system	148	14.6
	Strengthen my immune system	442	43.5
	It will neither strengthen nor weaken my immune system	369	36.4
	Weaken my immune system	45	4.4
	Greatly weaken my immune system	11	1.1
J11. Taking the COVID-19 vaccine:	Will give me complete freedom to get on with life just as before	270	26.6
	Will give me greater freedom	299	29.5
	Will have no effect on my freedom	406	40.0
	Will restrict my freedom	27	2.7
	Will completely restrict my freedom to get on with life	13	1.3
J12. I expect that receiving the vaccine will be:	Hardly noticeable	207	20.4
	A little unpleasant	689	67.9
	Moderately unpleasant	82	8.1

	Painful	24	2.4
	Extremely painful	13	1.3
J13. The side effects for people of getting the COVID-19 vaccine will be:	None	272	26.8
	Mild	651	64.1
	Moderate	57	5.6
	Significant	23	2.3
	Life-threatening	12	1.2
J14. Taking a new COVID-19 vaccine will make me feel like a guinea pig:	Do not agree	419	41.3
	Agree a little	246	24.2
	Agree moderately	287	28.3
	Agree a lot	49	4.8
	Completely agree	14	1.4
